# Antinuclear antibody targets in autoimmune hepatitis and drug-induced liver injury: Diagnostic relevance of nucleosome antibodies

**DOI:** 10.1016/j.jtauto.2026.100354

**Published:** 2026-01-29

**Authors:** Mirjam Kolev, Michèle Freiburghaus, Guido Stirnimann, Henning Nilius, Martin Wartenberg, Juliette Schlatter, Michael Nagler, Michael P. Horn, Nasser Semmo

**Affiliations:** a Department of Visceral Surgery and Medicine, Inselspital, Bern University Hospital, University of Bern, Switzerland; b Department of Clinical Chemistry, Inselspital, Bern University Hospital, University of Bern, Switzerland; c Department for BioMedical Research, Visceral Surgery and Medicine, University of Bern, Switzerland; d Institute of Tissue Medicine and Pathology, University of Bern, Switzerland; e Graduate School for Health Sciences, University of Bern, Switzerland; f University of Bern, Bern, Switzerland

**Keywords:** Autoimmune hepatitis, Drug-induced liver injury, Anti-nuclear antibodies, Anti-nucleosome antibodies, Anti-histone antibodies

## Abstract

**Background & Aims:**

Anti-nuclear antibodies (ANA) are detected in 70–100 % of patients with autoimmune hepatitis (AIH) and in 22–33 % of patients with drug-induced liver injury (DILI). However, little is known about the specific antigens which they bind to. This study aimed to investigate the antigen specificity of ANA in patients with AIH and DILI and to assess the diagnostic value of these antibodies in differentiating between the two diseases.

**Methods:**

We performed a retrospective, cross-sectional analysis of ANA-positive patients with AIH or DILI treated at a tertiary referral center. ANA patterns were determined by indirect immunofluorescence, followed by antigen-specific characterization using ELISA.

**Results:**

A total of 81 patients were included (54 AIH, 27 DILI). A homogeneous ANA pattern was more frequently observed in AIH (75.9 %) than in DILI (48.1 %). Among patients with a homogeneous pattern, anti-nucleosome antibodies were present in 51.2 % of AIH patients and 15.4 % of DILI patients, yielding a specificity of 84.6 % (95 % confidence interval (CI) 54.6–98.1) and a sensitivity of 51.2 % (95 % CI: 35.1–67.1). Anti-nucleosome antibodies demonstrated the highest diagnostic accuracy in this subgroup (area under the curve (AUROC): 0.85; 95 % CI: 0.70–1.00), outperforming or equaling Immunoglobulin G (IgG) (AUROC: 0.85; 95 % CI: 0.74, 0.95), anti-F-actin antibodies (AUROC: 0.81; 95 % CI: 0.69, 0.93) and anti-smooth muscle antibodies (SMA) (AUROC: 0.80; 95 % CI: 0.67, 0.92).

**Conclusions:**

In ANA-positive patients with a homogeneous ANA pattern, anti-nucleosome antibodies provide high diagnostic accuracy in distinguishing AIH from DILI. These findings suggest that anti-nucleosome antibodies may aid in the diagnostic workup of ANA-positive liver injury.

## Highlights

• Autoantibodies are an important hallmark in the diagnosis of autoimmune hepatitis (AIH).
• ANA are positive in a notable proportion of patients with drug-induced liver injury (DILI).
• Anti-nucleosome antibodies are significantly more prevalent in AIH than in DILI.
• Anti-nucleosome antibodies may serve as a useful tool to differentiate AIH from DILI.

## Teaser

Lay summary: Autoimmune hepatitis (AIH) is a condition in which the immune system mistakenly attacks the liver and typically requires long-term immunosuppressive therapy. In contrast, drug-induced liver injury (DILI) is caused by a reaction to a medication and usually improves after discontinuation of the offending drug, without the need of immunosuppression. Differentiating between these two liver diseases can be challenging, as they often present with similar features. In our study, we investigated whether the in-depth analysis of antinuclear antibodies (ANA) could help distinguish between AIH and DILI. We found that anti-nucleosome antibodies were clearly more frequently detected in patients with AIH than in those with DILI.

## 1 Introduction

Autoimmune hepatitis (AIH) and drug-induced liver injury (DILI) represent two major causes of hepatocellular inflammation with overlapping clinical, biochemical, and histological features, often posing a diagnostic challenge in clinical hepatology [1,2]. AIH is a chronic, immune-mediated liver disease characterized by interface hepatitis, elevated transaminases, elevated immunoglobulin G (IgG), and the presence of autoantibodies [3]. If left untreated, AIH can progress to cirrhosis and liver failure, but generally responds well to immunosuppressive therapy [4].

In contrast, DILI encompasses a heterogeneous group of liver injuries caused by prescription medications, over-the-counter drugs, or herbal and dietary supplements. DILI may mimic nearly all liver diseases, including AIH, both in clinical presentation and histopathology [1].

Antinuclear antibodies (ANA) are frequently detected in AIH [5], but their antigen specificity and clinical significance remain incompletely understood. Previous studies have described antibodies directed against nucleosomes [6], histones [7,8], centromeres, ribonucleoproteins, Ro60/TROVE2 and Ro52/TRIM21 [9] and single- or double-stranded DNA [10], though the literature on antigen specificity in AIH remains limited. ANA are also often detected in DILI, further complicating the diagnostic work-up [11,12]. In addition to serology, liver histology is a key component in the diagnostic evaluation. Hallmark histological features of AIH include dense portal lymphoplasmacytic infiltrates, interface hepatitis, lobular inflammation, and focal necrosis - findings typically more prominent in AIH than in DILI [[13], [14], [15], [16]].

Accurate distinction between AIH and DILI is clinically important, as the management strategies differ significantly. While DILI generally resolves after withdrawal of the causative agent and requires no immunosuppression, AIH requires long-term immunosuppressive therapy to prevent progression and relapse. However, the lack of disease-specific markers hampers diagnostic clarity and contributes to uncertainty in classification.

In this study, we investigated the antigenic specificity of ANA in patients with AIH and DILI using indirect immunofluorescence and ELISA-based assays. We also examined conventional serological and histological parameters, aiming to define distinguishing features that could enhance diagnostic precision and guide clinical management. With this work, we seek to contribute to the refined classification of immune-mediated versus drug-induced liver injury by evaluating the diagnostic value of specific ANA subtypes.

## 2 Patients and methods

### 2.1 Patients and samples

This is a retrospective, cross-sectional, monocentric study performed at the University Hospital of Bern, a tertiary care center in Switzerland. Adult patients who received medical care between January 01, 2011 and December 31, 2022 were eligible if they had a diagnosis of AIH or DILI, tested positive for ANA at the time of diagnosis and/or sample collection, had provided written general informed consent, and had no other concomitant chronic liver disease. The author extracting clinical data was able to identify individual participants; however, the data were anonymized prior to analysis. Diagnosis of AIH was based on the simplified criteria of the International AIH group [17]. Patients with a score of ≥5 points were classified as having probable or definite AIH, provided that the clinical context and treatment response (e.g. biochemical improvement under corticosteroids) supported the diagnosis. Histological confirmation was mandatory for all AIH patients. None of the patients had an overlap syndrome with other autoimmune liver diseases. DILI was diagnosed using the Roussel Uclaf Causality Assessment Method (RUCAM), and only cases classified as ‘probable’ or higher were included (score ≥6) [18]. In addition, spontaneous biochemical improvement following drug withdrawal was required to confirm the diagnosis. Available liver biopsies were reviewed by an experienced liver pathologist. Histological grading and staging were assessed using the Ishak scoring system [19], and fibrosis was further staged according to the METAVIR classification [20]. Remission in AIH was defined as normalization of ALT and IgG.

Samples were obtained from the Hepatology Biobank of the University Hospital Bern, Switzerland. ANA testing was initially performed as part of routine clinical diagnostics at the time of sample collection. For the purpose of this study, ANA testing and pattern assignment were repeated in all available samples using current laboratory standards, and staining patterns were classified according to the International Consensus on Antinuclear Antibody Patterns (ICAP) nomenclature.

### 2.2 Laboratory methods

ANA testing was performed using indirect immunofluorescence (IIF) on HEp2-cells (Werfen/INOVA Diagnostics, San Diego) according to manufacturer's instructions. Samples with ANA titers ≥1:80 were further analyzed for specific antibody patterns using antigen-specific immunoassays, in accordance with the International Consensus on Antinuclear Antibody Patterns [21]. In cases displaying a homogeneous nuclear pattern (AC-1), additional testing for anti–double-stranded DNA (anti-dsDNA using plasmid DNA as source of antigen; EliA, Phadia Thermo Fisher, Uppsala, Sweden), anti-nucleosomes and anti-histone antibodies was performed using commercial IVD-CE labelled ELISA kits (both from Werfen/INOVA Dx, San Diego). Samples showing a fine speckled nuclear pattern (AC-4) without concurrent AC-1 were analyzed using the Symphony^*S*^ multiplex assay (EliA, Phadia Thermo Fisher, Uppsala, Sweden). Equivocal or positive Symphony^*S*^ results were followed by confirmatory testing for individual target antigens, including Ro60/TROVE2, Ro52/TRIM21, SS-B (La), U1RNP (A,C,70 kDa), SmD_3_, CENP-B, Scl-70 and Jo-1). Weak cytoplasmic patterns (AC-19/20) were not subjected to further investigation. One AIH patient with a distinct centromere-pattern (AC-3) was confirmed to have anti–centromere antibodies using the anti-CENP-EliA assay rather than the Symphony^S^ panel.

The anti-nucleosome ELISA (Werfen/INOVA) is based on stripped chromatin preparations lacking histone H1 and high-mobility group proteins. Highly purified chromatin from calf thymus consisting of DNA surrounding the core histone octamers (H2A-H2B-H3-H4). During purification, the H1 histones and non-histone proteins were removed. The anti-histone ELISA (Werfen/INOVA) is based on affinity-chromatographically purified histone antigens bound to the wells of the polystyrene microtiter plate while maintaining their original configuration.

### 2.3 Statistical analysis

Categorical variables are expressed as absolute numbers and percentages. Continuous variables are reported as either medians with interquartile range (25IQR-75IQR) or means with standard deviations, as appropriate. Comparisons of categorical variables between groups were performed using the chi-square test or Fisher's exact test, where expected frequencies were less than five. For comparisons of continuous variables, the Kruskal-Wallis test was used.

All ANA-positive patients with AIH or DILI were included in the study. For subgroup analyses based on ANA pattern, patients were stratified into AC-1 (homogeneous) positive and AC-1 negative groups. Only one patient with an isolated AC-3 pattern was excluded from the AC-1 positive versus AC-1 negative analysis. To evaluate the diagnostic performance of serological tests in differentiating AIH from DILI, we calculated sensitivity, specificity, positive and negative likelihood ratios, and the area under the receiver operating characteristic curve (AUROC). These metrics were assessed separately for the AC-1 positive and AC-1 negative groups using the R packages epiR and pROC (R Version 4.1.2).

P values < 0.05 (two-tailed) were considered statistically significant in all analysis.

Missing clinical data were assumed to be missing at random and are explicitly indicated in the results section.

### 2.4 Ethical approval

The study was approved by the Cantonal Ethics Commission of Bern, Switzerland (2020-01517) and conducted according to the Declaration of Helsinki.

## 3 Results

### 3.1 Patient characteristics

#### 3.1.1 Study inclusion

**Fig. 1:**
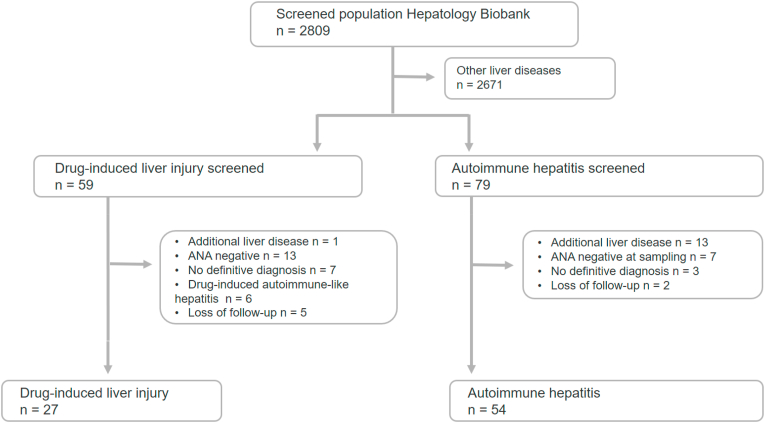
Study flow chart for patient identification.

The study flowchart (Fig. 1) illustrates the screening and enrollment process. From a total of 2809 patients in the Hepatology Biobank, 27 patients with DILI and 54 patients with AIH fulfilled the eligibility criteria and were included in the final analysis. Samples were obtained at the time of diagnosis or within one month thereafter in 23 (85.2 %) patients with DILI and in 37 (69 %) patients with AIH. In cases where samples were collected during follow-up, ANA titers were consistently elevated, including at the time point of sampling. Among these, the median time from diagnosis to sample collection was 88 days (IQR 70–113) in the DILI group and 1061 days (IQR 288–3483) in the AIH group.

#### 3.1.2 Baseline characteristics

Table 1 summarizes the main demographic and laboratory features of the study population. Among patients with DILI, 22 (81.5 %) had a “probable” and five (18.5 %) a “highly likely” diagnosis according to the RUCAM score. Patients with DILI were significantly more likely to present with a cholestatic pattern of liver injury (p = 0.027), whereas patients with AIH more commonly exhibited a hepatocellular injury pattern (p = 0.010), along with higher levels of alanine aminotransferase (ALT) (p = 0.022) and international normalized ratio (INR) (p = 0.001). In contrast, levels of alkaline phosphatase and bilirubin did not significantly differ between the two groups. IgG levels, ANA titers, as well as the frequency of SMA and anti-actin positivity were significantly higher in AIH patients compared to those with DILI. No significant differences were observed between groups with respect to liver stiffness measurements.

**Table 1:** Baseline characteristics of patients with drug-induced liver injury and autoimmune hepatitis at diagnosis.

	DILI n = 27	AIH n = 54	Missing values DILI/AIH	p-value
Demographics				
Female sex, n (%)	17 (63.0)	40 (74.1)		0.439
Age at diagnosis, years	60 (40–66)	57 (44–68)		0.627
Caucasian, n (%)	23 (85.2)	50 (92.6)		0.510
Body mass index, kg/m^2^	24.2 (22.5–28.2)	24.6 (22.0–28.6)		0.991
Biochemistry				
ALT, x ULN	9.4 (3.6–19.2)	17.5 (9.5–29.5)	0 (0)/6 (11.1)	0.022∗
Alkaline phosphatase, x ULN	1.39 (1.09–1.87)	1.37 (0.91–1.99)	0 (0)/6 (11.1)	0.430
Bilirubin total, μmol/l (<17 μmol/l)	54 (20–130)	29 (17–144)	0 (0)/6 (11.1)	0.643
INR (<1.2)	1.07 (1.00–1.19)	1.20 (1.09–1.42)	0 (0)/8 (14.8)	0.001∗
Immunoglobulin G, g/l (7–16 g/L)	10.7 (10.0–14.9)	22.3 (14.7–31.4)	3 (11.1)/8 (14.8)	<0.001∗
Hypereosinophilia, present (>0.4 G/L)	8 (36.4)	14 (25.9)	5 (18.5)/6 (11.1)	0.528
Pattern of liver injury				
Hepatocellular, n (%)	15 (55.6)	41 (85.4)	0 (0)/6 (11.1)	0.010∗
Mixed, n (%)	8 (29.6)	7 (14.6)	0 (0)/6 (11.1)	0.207
Cholestatic, n (%)	4 (14.8)	0 (0.0)	0 (0)/6 (11.1)	0.027∗
Autoimmune serology				
ANA, Titer ≥1:80	27 (100)	54 (100)		1.00
ANA, Titer ≥1:160	15 (55.6)	46 (85.2)		0.008∗
ANA, Titer ≥1:320	7 (25.9)	45 (83.3)		<0.001∗
ANA, Titer ≥1:640	4 (14.8)	30 (55.6)		0.001∗
ANA, Titer ≥1:1280	2 (7.4)	22 (40.7)		0.005∗
SMA, Titer ≥1:80	6 (22.2)	42 (84)	0 (0)/2 (3.7)	<0.001∗
F-Actin, ≥20 Units	3 (11.1)	41 (78.8)	0 (0)/2 (3.7)	<0.001∗
LKM-1, Titer ≥1:80 or p450 ≥ 20 Units	0 (0.0)	3 (5.8)	0 (0)/2 (3.7)	1.00
SLA, ≥20 Units	1 (3.7)	6 (11.5)	0 (0)/2 (3.7)	0.456
Fibroscan, kPa	10.7 (7.5–15.4)	13.0 (8.2–28.8)	8 (29.6)/28 (51.9)	0.255
Presence of other autoimmune diseases, n (%)	11 (40.7)	21 (38.9)		0.872

Categorical variables presented as n (%). Continuous data presented as median and interquartile range (Q25-Q75). The pattern of liver injury was defined by the R-value; (ALT/ULN) ÷ (Alk Phos/ULN). Cases were classified as hepatocellular (R ≥ 5), cholestatic (R ≤ 2) or mixed (R > 2 and R < 5) [22]. ∗ indicates a significant p-value.

AIH, autoimmune hepatitis; ALT, alanine aminotransferase; Alk Phos, alkaline phosphatase; ANA, anti-nuclear-antibodies; DILI, drug-induced liver injury; IgG, immunoglobulin G; INR, international normalized ratio; LKM-1, liver-kidney-microsomal antibodies; SLA, soluble liver antigen antibodies; SMA, smooth-muscle cell antibodies; ULN, upper limit of normal.

#### 3.1.3 Causative drugs

An overview on the causative drug classes in patients with DILI is shown in Table 2. Among DILI cases, antibiotics represented the most frequently identified causative drug group. None of the patients reported prior use of anabolic steroids, herbal remedies, or dietary supplements. A comprehensive list of the causative drugs is provided in Supplementary Table 1.

**Table 2:** Categories of drugs implicated in drug-induced liver injury.

Drug categories	Drug-induced liver injury n = 27
Analgesics, n (%)	3 (11.1)
Antibiotics, n (%)	11 (40.8)
Statins, n (%)	2 (7.4)
Immunosuppressants, n (%)	3 (11.1)
Others, n (%)	2 (7.4)
Unclassifiable, n (%)	6 (22.2)

‘Other’ drugs consisted of sartans and psychotropic drugs.

If the category of the causative drug was unclear because of the intake of several drugs, the patient was categorized as ‘unclassifiable’.

#### 3.1.4 Liver histology

All patients diagnosed with AIH underwent liver biopsy. Of these, 46 biopsies (85.1 %) were successfully reevaluated by an experienced liver pathologist. For an additional seven AIH patients (12.9 %), in whom original histological slides were unavailable, detailed histology reports were reviewed, allowing inclusion in the assessment. Thus, liver histology was available or assessable in 98.1 % of AIH cases. Among patients with DILI, 22 of 27 (81.5 %) underwent liver biopsy, and all corresponding specimens were available for reevaluation. The median interval between drug exposure and either histological evaluation or initial presentation at our center was 62.5 days (IQR 31–103). Detailed histological findings are summarized in Table 3.

**Table 3:** Overview on the findings from liver biopsies of patients with drug-induced liver injury and autoimmune hepatitis at diagnosis.

	DILI n = 27	AIH n = 54	Missing values DILI/AIH	p-value
Inflammation				
Lobular inflammation, n (%)	15 (68.2)	53 (100)	5 (18.5)/1(1.85)	<0.001∗
Interface hepatitis, n (%)	11 (50)	49 (92.5)	5 (18.5)/0(0)	<0.001∗
Portal lymphoplasmacytic infiltrate, n (%)	6 (27.3)	52 (96.3)	5 (18.5)/0(0)	<0.001∗
Presence of grouped plasma cells, n (%)	6 (27.3)	41 (87.2)	5 (18.5)/7(13)	<0.001∗
Eosinophilic infiltrate, n (%)	12 (54.5)	26 (49.1)	5 (18.5)/1(1.85)	0.858
Feathery degeneration, n (%)	3 (13.6)	29 (55.8)	5 (18.5)/2(3.7)	0.002∗
Fibrous collapse, n (%)	12 (54.5)	34 (64.2)	5 (18.5)/1(1.85)	0.605
Periportal or periseptal interface hepatitis (Category A)			5 (18.5)/1(1.85)	<0.001∗
Absent interface hepatitis, n (%)	11 (50)	2 (3.8)		<0.001∗
Mild interface hepatitis, n (%)	8 (36.4)	10 (18.9)		0.187
Mild to moderate interface hepatitis, n (%)	0 (0)	9 (17)		0.095
Moderate hepatitis, n (%)	1 (4.5)	10 (18.9)		0.216
Severe interface hepatitis, n (%)	2 (9.1)	22 (41.5)		0.014∗
Confluent necrosis (Category B)			5 (18.5)/1(1.85)	0.273
Absent confluent necrosis, n (%)	12 (54.5)	18 (34)		0.162
Focal confluent necrosis, n (%)	3 (13.6)	6 (11.3)		1.000
Zone 3 necrosis in some areas, n (%)	2 (9.1)	13 (24.5)		0.228
Zone 3 necrosis in most areas, n (%)	2 (9.1)	4 (7.5)		1.000
Zone 3 necrosis and occasional portal-central bridging, n (%)	0 (0)	6 (11.3)		0.239
Zone 3 necrosis and multiple portal-central bridging, n (%)	3 (13.6)	4 (7.5)		0.697
Panacinar or multiacinar necrosis, n (%)	0 (0)	2 (3.8)		0.891
Focal spotty lytic necrosis, apoptosis and focal inflammation (Category C)			5 (18.5)/1(1.85)	0.004∗
Absent focal necrosis per 10×objective, n (%)	7 (31.8)	2 (3.8)		0.003∗
One focus or less per 10×objective, n (%)	6 (27.3)	12 (23.1)		0.930
Two to four foci per 10×objective, n (%)	4 (18.2)	11 (21.2)		1.000
Five to ten foci per 10×objective, n (%)	2 (9.1)	22 (42.3)		0.012∗
More than ten foci per 10×objective, n (%)	3 (13.6)	5 (9.6)		0.912
Portal inflammation (Category D)			5 (18.5)/2(3.7)	<0.001∗
No portal inflammation, n (%)	3 (13.6)	0 (0)		0.036∗
Mild portal inflammation in some or all portal areas, n (%)	8 (36.4)	3 (5.7)		0.002∗
Moderate portal infiltrate in some or all portal areas, n (%)	9 (40.9)	16 (30.2)		0.530
Moderate/marked portal inflammation in all portal areas, n (%)	1 (4.5)	23 (43.4)		0.003∗
Marked portal inflammation in all portal areas, n (%)	1 (4.5)	11 (20.8)		0.162
Fibrosis staging			5 (18.5)/8(14.8)	0.055
No fibrosis, n (%)	17 (77.3)	19 (41.3)		<0.001∗
Fibrous expansion of some portal areas with or without short fibrous septa, n (%)	5 (22.7)	9 (19.6)		1.000
Fibrous expansion of most portal areas, with or without short fibrous septa, n (%)	0 (0)	5 (10.9)		0.267
Fibrous expansion of most portal areas with occasional portal to portal bridging, n (%)	0 (0)	5 (10.9)		0.267
Fibrous expansion of portal areas with marked bridging as well as portal to central, n (%)	0 (0)	4 (8.7)		0.382
Marked bridging, n (%)	0 (0)	3 (6.5)		0.553
Cirrhosis, n (%)	0 (0)	1 (2.2)		1.000
Fibrosis			5 (18.5)/8(14.8)	0.016∗
METAVIR 0–1, n (%)	22 (100)	29 (64.4)		0.004∗
METAVIR 2, n (%)	0 (0)	6 (13.3)		0.180
METAVIR 3–4, n (%)	0 (0)	9 (20)		0.061

Liver biopsies were evaluated according to the Ishak Score [11]. Fibrosis was furthermore classified according to the METAVIR scoring system [12]. ∗ indicates a significant p-value.

AIH, autoimmune hepatitis; DILI, drug-induced liver injury.

Features such as lobular inflammation, portal lymphoplasmacytic infiltrates, grouped plasma cells, interface hepatitis, and feathery degeneration were observed significantly more frequently in patients with AIH compared to those with DILI. Notably, the absence of interface hepatitis was significantly more common in DILI (50 %, n = 11) than in AIH (3.8 %, n = 2) (p < 0.001), whereas severe interface hepatitis was observed in 41.5 % (n = 22) of AIH cases but only in 9.1 % (n = 2) of DILI patients (p = 0.002). Portal inflammation was significantly less pronounced in DILI (p < 0.001), and focal spotty necrosis was more frequently observed in AIH (p = 0.004). In contrast, confluent necrosis and fibrous collapse did not differ significantly between groups. Fibrosis was significantly more common among AIH patients compared to DILI (p < 0.001). While moderate to advanced stages of fibrosis occurred exclusively in AIH, this difference did not reach statistical significance.

#### 3.1.5 Outcomes

Among the DILI cohort, one progressed to chronic progressive hepatopathy following an initial episode of acute hepatitis, while the other patients achieved resolution of their liver injury. In contrast, 77.6 % of those with AIH attained remission within one year of diagnosis. Notably, the median duration of immunosuppressive therapy spanned 72 (40.5–114) months, for AIH patients. At the last follow-up, a substantial majority of AIH patients (83.3 %, n = 45) were continuing immunosuppression. Complications were observed in the AIH group, where one patient necessitated liver transplantation eight months post-diagnosis, and another succumbed to infectious complications during the initial treatment phase. There were no fatalities or transplantations reported among the DILI patients.

### 3.2 ANA patterns observed in indirect immunofluorescence and antibodies measured with enzyme-linked immunosorbent assays

**Fig. 2:**
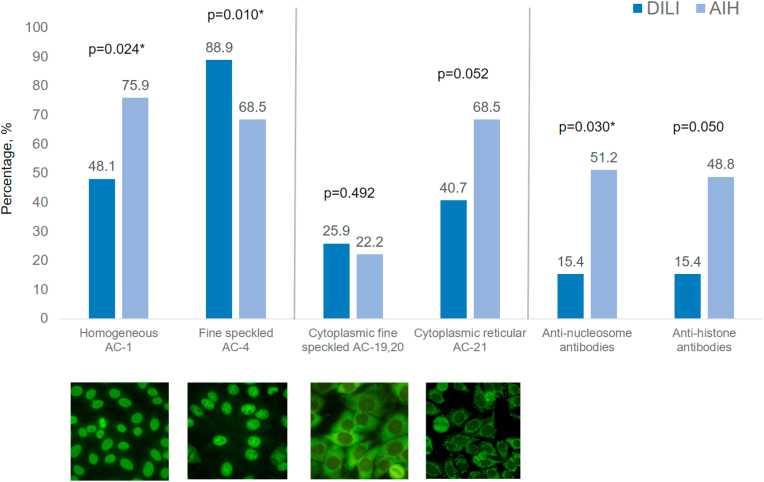
Most relevant nuclear and cytoplasmic ANA patterns and most relevant antibodies measured with ELISA. ∗ indicates a significant p-value. The use of antibody pattern images from the International Consensus on ANA Patterns was approved, and permission was obtained prior to publication. AC, anti-cell; AIH, autoimmune hepatitis; DI-ALH, drug-induced AIH-like hepatitis; DILI, drug-induced liver injury.

Table 4 provides a detailed overview of the observed ANA patterns and the corresponding antibodies, with key findings graphically summarized in Fig. 2.

**Table 4:** Overview on main ANA patterns observed in indirect immunofluorescence and antibodies measured with immunoassays. Indentation shows that these tests were only performed if the test one level above was positive.

Test	DILI n (%)	AIH n (%)	p-value
**N Total**	**27**	**54**	
**AC-1**	**13 (48.1)**	**41 (75.9)**	**0.0**2^a^
dsDNA	1 (7.7)	4 (9.8)	1.00^b^
Nucleosomes	2 (15.4)	21 (51.2)	0.03^a^,^b^
Histones	2 (15.4)	20 (48.71)	0.05^b^
**AC-4**	**14 (51.9)**	**12 (22.2)**	0.01^a^
Symphony^*S*^*equivocal*	0 (0.0)	1 (8.3)	0.46^b^
Symphony^*S*^*positive*	0 (0.0)	3 (25.0)	0.08^b^
Ro60/TROVE2	n.a.	1 (25.0)	–
SS-B/La	n.a.	1 (25.0)	–
Ro52/TRIM21	n.a.	3 (75.0)	–
**AC-3**	**0 (0.0)**	**1 (1.9)**	**1.00**^b^
CENP-B	n.a.	1 (100.0)	–

Pattern-specific antibody testing with ELISA were performed if ANA titers were ≥1:80.

Symphony^*S*^*equivocal* results are those between 0.7 and 1.0, which the manufacturer defines as a gray area. However, in that range, we still differentiated the antibodies.

AC, anti-cell; AIH, autoimmune hepatitis; Ro60/TROVE2, Ro60 autoantigen (gene TROVE2); Ro52/TRIM21, Ro52 autoantigen (TRIM21 gene/protein); dsDNA, double stranded deoxyribonucleic acid; CENP-A/B, centromere antibodies; SS-B, SS-B (La) antibodies; n.a., not applicable.

a Indicates a significant p-value.

b Fisher's exact test was used.

The most frequently observed nuclear patterns were homogeneous (AC-1) and fine speckled (AC-4). A homogeneous ANA pattern (AC-1) was significantly more prevalent among patients with AIH, occurring in 41 of 54 cases (75.9 %), compared to 13 of 27 patients with DILI (48.1 %) (p = 0.024). Conversely, the fine speckled pattern (AC-4) was more commonly seen in DILI patients (88.9 %, n = 24) than in AIH (68.5 %, n = 37) (p = 0.010). Other nuclear patterns were very rare; only one patient exhibited a different pattern (AC-3), as detailed in Table 4.

Cytoplasmic ANA patterns were present in 63 % of DILI patients (n = 17) and 79.6 % of AIH patients (n = 43) (p = 0.180). The most frequent cytoplasmic pattern was the reticular/mitochondrial pattern (AC-21), observed in 40.7 % of DILI patients (n = 11) and 68.5 % of AIH patients (n = 37) (p = 0.052). A cytoplasmic fine speckled pattern (AC 19–20) was identified in 25.9 % of DILI (n = 7) and 22.2 % of AIH patients (n = 12) (p = 0.492). Most cytoplasmic staining patterns were only weakly positive, limiting further differentiation.

In the subgroup of patients with ANA titers ≥1:80 and a homogeneous pattern (AC-1) ELISA-based antibody profiling revealed that anti-nucleosome antibodies were significantly more prevalent in AIH patients (51.2 %, n = 21) than in DILI patients (15.4 %, n = 2) (p = 0.030). A similar trend was observed for anti-histone antibodies, which were positive in 48.8 % AIH patients (n = 20) compared to 15.4 % of DILI patients (n = 2) (p = 0.050).

### 3.3 Diagnostic performance of laboratory tests in subgroups

Among patients with a homogeneous ANA pattern (AC-1) (n = 54; AIH: n = 41, DILI: n = 13), anti-nucleosome antibodies demonstrated the highest diagnostic accuracy for distinguishing AIH from DILI, with an AUROC of 0.85 (95 % CI: 0.70–1.00). At the manufacturer's recommended cut-off of 20 U/mL, 21 of 41 AIH patients tested positive (sensitivity: 51.2 %; 95 % CI: 35.1–67.1 %), compared to only 2 of 13 DILI patients (specificity: 84.6 %; 95 % CI: 54.6–98.1 %). Lowering the cut-off to 5 U/ml, based on Youden's index, increased sensitivity to 87.8 % (95 % CI: 73.8–96.0 %), while specificity remained unchanged, as no additional DILI patients tested positive at this cut-off.

**Fig. 3:**
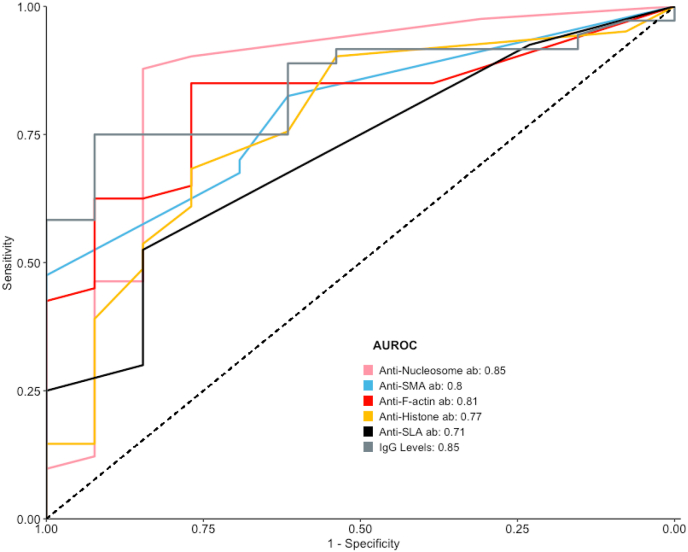
Receiver operating characteristic curve for patients with AC-1 ANA. ab, antibodies; AC, anti-cell; ANA, anti-nuclear antibodies; AUC, area under the curve; SLA, soluble liver antigen; LKM, liver-kidney microsomal 1 (p450).

In comparison, other diagnostic markers in this subgroup showed similar or slightly lower diagnostic performance (Fig. 3): IgG levels (AUROC: 0.85; 95 % CI: 0.74–0.95), anti-F-actin antibodies (AUROC: 0.81; 95 % CI: 0.69–0.93), anti-SMA antibodies (AUROC: 0.80; 95 % CI: 0.67–0.92), anti-histone antibodies (AUROC: 0.77; 95 % CI: 0.61–0.92), anti-SLA antibodies (AUROC: 0.71; 95 % CI: 0.57–0.85), anti-dsDNA antibodies (AUROC: 0.66; 95 % CI: 0.49–0.83), and anti-LKM1 antibodies (AUROC: 0.46; 95 % CI: 0.41–0.50).

In patients without a homogeneous ANA pattern (n = 27; AIH: n = 13, DILI: n = 14), ELISA-based screening using the Phadia Symphony^*S*^ panel identified three AIH cases. These cases were positive for antibodies included in the broad ANA/cytoplasmic panel, although these specificities are not AIH-specific. The sensitivity of this approach was 25.0 % (95 % CI: 5.0–57.0 %) with a specificity of 100 % (95 % CI: 76.0–100.0 %), yielding an AUROC of 0.80 (95 % CI: 0.60–0.99). In the same subgroup, conventional AIH-specific immunoassays showed superior diagnostic performance: Anti-F-actin antibodies: AUROC 0.91 (95 % CI: 0.79–1.00), anti-SMA antibodies: AUROC 0.86 (95 % CI: 0.73–1.00), IgG: AUROC 0.83 (95 % CI: 0.62–1.00).

## 4 Discussion

Accurately distinguishing between DILI and AIH is essential for guiding treatment decisions, particularly regarding the initiation or avoidance of immunosuppressive therapy. In this study, we investigated ANA target antigens in both conditions to assess their potential diagnostic value.

The most notable result of our study is the marked difference in anti-nucleosome antibody prevalence between AIH and DILI patients with a homogeneous (AC-1) ANA pattern. Anti-nucleosome antibodies were significantly more common in AIH and demonstrated the highest diagnostic accuracy among all tested serological markers. This finding is in line with previous research by Yokokawa et al. [6], who reported increased levels of these antibodies in AIH compared to chronic viral hepatitis and healthy controls. To our knowledge, our study is the first to demonstrate the diagnostic utility of ANA antigen differentiation - particularly anti-nucleosome antibodies - in distinguishing AIH from DILI. Although 15.4 % of DILI patients were anti-nucleosome positive using standard cutoffs, anti-nucleosome antibodies still showed high specificity in ANA-positive patients with a homogeneous pattern, supporting their potential role in distinguishing AIH from DILI. Nevertheless, this overlap indicates that anti-nucleosome antibodies should be interpreted alongside other clinical and laboratory findings. This is of special relevance since current guidelines do not recommend routine ANA subtyping due to previously assumed limited clinical relevance [23].

Anti-histone antibodies were also more prevalent in AIH, although the difference did not reach statistical significance. The observed frequencies in our AIH cohort are comparable to previous reports by Czaja et al. [8] and Chen et al. [7]. However, their lower diagnostic accuracy reflects limited discriminatory power, particularly in cases where clinical differentiation between AIH and DILI is most challenging. These observations are in line with findings by Lammert et al. [24], who also questioned the utility of anti-histone antibodies in differentiating liver disease phenotypes. Unlike earlier studies, our cohort showed a low prevalence of anti-dsDNA antibodies [10], suggesting a limited role for these markers in distinguishing AIH from DILI in clinical practice. Differences in assay methodology may partly explain these discrepancies, as our study used an ELISA based on plasmid DNA, whereas Czaja et al. [10] also applied the Crithidia luciliae immunofluorescence test, which is known to detect only high-affinity anti-dsDNA antibodies. In contrast, anti-nucleosome ELISA may also detect antibodies directed against conformational epitopes consisting of DNA and histone proteins.

These findings suggest that anti-nucleosome antibodies are particularly valuable for differentiating AIH from DILI in patients with a homogeneous ANA pattern, while traditional autoantibody assays such as anti-SMA and anti-F-actin remain helpful in patients with non-homogeneous patterns. This highlights the potential for a stratified serological approach based on ANA pattern to optimize diagnostic accuracy.

Our findings confirm that patients with AIH exhibit significantly higher ANA titers, serum IgG levels, and rates of SMA and anti-F-actin antibody positivity compared to patients with DILI. These results underscore the continued relevance of these established biomarkers in differentiating AIH from DILI [25].

The most commonly implicated drugs in our DILI cohort were amoxicillin-clavulanate, non-steroidal anti-inflammatory drugs (NSAIDs; e.g., ibuprofen, diclofenac), and atorvastatin. These agents are well recognized in the literature as frequent causes of DILI [1,26], reinforcing the generalizability and clinical relevance of our cohort.

Histological assessment supported the distinction between the two disease entities. In line with previous studies [[13], [14], [15], [16]], AIH patients demonstrated more pronounced portal lymphoplasmacytic infiltrates, grouped plasma cells, interface hepatitis, lobular inflammation, and focal necrosis. In contrast, DILI cases were more likely to show absent or mild fibrosis, reflecting the typically acute and self-limiting course of DILI compared to the chronic inflammatory nature of AIH. Regarding ANA patterns, homogeneous (AC-1) and fine speckled (AC-4) nuclear staining were the most prevalent in both groups. However, the homogeneous pattern was significantly more frequent among AIH patients - a finding consistent with earlier reports [23]. Cytoplasmic staining patterns were observed in both cohorts but were predominantly of low density and showed no disease-specific associations. Anti-mitochondrial antibodies were not performed systematically, as clinical and biochemical features of primary biliary cholangitis were absent.

Taken together, our findings reveal that ANA target specificity differs significantly between AIH and DILI. Anti-nucleosome antibodies, in particular, emerged as a promising biomarker with high specificity and sensitivity for AIH among patients presenting with a homogeneous ANA pattern.

### 4.1 Strengths and limitations of the study

This study has several limitations that merit consideration. A limitation of this study is the relatively small sample size, which may reduce statistical power and limit the generalizability of the findings. Its retrospective design inherently carries the risk of heterogeneity in diagnostic workup and missing data, which may influence the interpretation of our results. Additionally, in approximately 30 % of patients with AIH, sample acquisition occurred during follow-up rather than at the time of diagnosis. While this could potentially affect the reproducibility of serological findings, all of these patients had persistently elevated ANA titers throughout the observation period, supporting their inclusion in the analysis. Additionally, this study did not include specific testing for anti-ribosomal P antibodies. While cytoplasmic patterns on HEp-2 cells may occasionally be observed in anti-ribosomal P–positive sera, HEp-2 indirect immunofluorescence is known to have limited sensitivity for the detection of these antibodies [22]. Consequently, the absence of dedicated solid-phase assays (e.g., ELISA or bead-based immunoassays) may have led to an underestimation of anti-ribosomal P antibody prevalence, which should be considered when interpreting the serological findings.

Furthermore, the monocentric nature of our study may limit the generalizability of the results, as it does not fully reflect the variability in clinical presentations, treatment responses, and diagnostic practices encountered in broader, more heterogeneous populations. These limitations underscore the need for prospective, multicenter studies with larger and more diverse patient cohorts to validate our findings. Such efforts will be crucial to refining the diagnostic approach and enhancing the accuracy of distinguishing AIH from DILI in clinical practice.

## 5 Conclusions

In this study, we confirmed the diagnostic value of high ANA titers, SMA and F-actin positivity, elevated IgG levels, and characteristic liver histology in distinguishing AIH from DILI. Importantly, among patients with a homogeneous ANA pattern, anti-nucleosome antibodies demonstrated the highest diagnostic accuracy for differentiating between these two conditions. These findings highlight the potential of targeted autoantibody profiling to improve diagnostic precision in challenging hepatological cases. Prospective studies in independent cohorts are needed to validate these results and clarify their clinical applicability.

## CRediT authorship contribution statement

**Mirjam Kolev:** Writing – review & editing, Writing – original draft, Visualization, Supervision, Project administration, Methodology, Formal analysis, Data curation, Conceptualization. **Michèle Freiburghaus:** Writing – review & editing, Writing – original draft, Data curation. **Guido Stirnimann:** Writing – review & editing, Visualization, Methodology, Conceptualization. **Henning Nilius:** Writing – review & editing, Writing – original draft, Visualization, Methodology, Formal analysis. **Martin Wartenberg:** Writing – review & editing, Data curation. **Juliette Schlatter:** Writing – review & editing, Data curation. **Michael Nagler:** Writing – review & editing, Resources, Methodology, Funding acquisition, Conceptualization. **Michael P. Horn:** Writing – review & editing, Writing – original draft, Visualization, Supervision, Resources, Project administration, Methodology, Formal analysis, Data curation, Conceptualization. **Nasser Semmo:** Writing – review & editing, Writing – original draft, Supervision, Project administration, Methodology, Funding acquisition, Conceptualization.

## Financial disclosure

MK has financial support from the Swiss 10.13039/501100012451Liver Foundation. The funding source had no influence in the writing of the report.

## Declaration of competing interest

The authors declare that they have no known competing financial interests or personal relationships that could have appeared to influence the work reported in this paper.

SN: Consultant of Gilead and Abbvie, has grant/research support from Gilead. HMP received speaking fees from RUWAG Handels AG, the provider of the INOVA reagents.

## Data Availability

Data will be made available on request.
