# Supplementary material for: Antinuclear antibody targets in autoimmune hepatitis and drug-induced liver injury: Diagnostic relevance of nucleosome antibodies
Source: J Transl Autoimmun. 2026 Jan 29;12:100354. doi: 10.1016/j.jtauto.2026.100354 (PMC12886071; doi:10.1016/j.jtauto.2026.100354)
Supplement: Multimedia component 1 [file mmc1.docx]

| **Drug-induced liver injury n=27** |
| --- |
| Co-amoxicillin (5) |
| Cefuroxime |
| Ceftriaxone, metronidazole, vancomycin |
| Clarythromycin, co-amoxicillin, diflucan, metronidazol |
| Ribociclib |
| Itroconazol, amoxicillin |
| Doxycycline |
| Terbinafine |
| Non-steroidal anti-inflammatory drugs, not specified (2) |
| Diclofenac |
| Atorvastatin (2) |
| Infliximab |
| Nivolumab |
| Leflunomid |
| Olmesartan |
| Sartan, protone-pump inhibitor |
| Quetiapin, sertralin, promazin, olanzapin, haloperidol, pregabalin, clonidin, pantoprazol, paracetamol, celecoxib |

**Supplementary Table 1** *Causative drugs in* *drug-induced liver injury.*

If drugs were causative in more than one patient, the number of patients is given in brackets. If several medications are possible for one patient, the drugs are listed in one cell.

DILI, drug-induced liver injury
